# Adverse childhood experiences do not moderate the association between aggressive antisocial behavior and general disinhibition in a forensic psychiatric inpatient sample

**DOI:** 10.3389/fpsyg.2022.1019246

**Published:** 2022-10-21

**Authors:** Adam Meddeb, Johan Berlin, Natalie Laporte, Märta Wallinius

**Affiliations:** ^1^Child and Adolescent Psychiatry, Department of Clinical Sciences Lund, Lund University, Lund, Sweden; ^2^Center of Ethics, Law and Mental Health, Institute of Neuroscience and Physiology, The Sahlgrenska Academy, University of Gothenburg, Gothenburg, Sweden; ^3^Department of Research, Regional Forensic Psychiatric Clinic, Växjö, Sweden

**Keywords:** adverse childhood experiences, childhood trauma, cycle of violence, aggressive antisocial behavior, offenders, forensic psychiatry, disinhibition, moderation

## Abstract

Adverse childhood experiences (ACE) and high levels of disinhibition have been associated with a variety of negative outcomes such as aggressive antisocial behavior (AAB). However, forensic psychiatric populations remain an understudied group in this field of research. This study aimed to fill that gap by investigating associations between ACE, AAB, and disinhibition in a forensic psychiatric sample. Furthermore, we aimed to explore such findings by investigating whether ACE might have a moderating effect on the association between disinhibition and AAB. A sample of forensic psychiatric patients (*n* = 89) was recruited from a high-security forensic psychiatric facility in Sweden. All study variables were moderately to strongly related to each other, although we found no moderating effect of ACE. *Post hoc* analysis indicated that our ACE items had differential effects on AAB scores, with placement outside the family home, absent parents, and parental drug abuse producing the largest effect on AAB levels. Our findings are in line with previous research demonstrating a significant and robust relationship between ACE, AAB, and disinhibition. Forensic psychiatric populations are exposed to high levels of both self-reported and documented ACE. This calls for trauma-informed care and highlights the importance of considering ACE in risk assessment, preventive work, and policy making.

## Introduction

Human suffering and economic burdens due to aggressive antisocial behaviors (AAB) present great challenges to societies. In the year 2000, an estimated 1.6 million people in the world lost their lives to violence (World Health Organization, 2002). However, lethal crime is only one measure of AAB and does not include minor offenses or aggressive and antisocial acts that escape the attention of the legal system, thus suggesting a significant number of unreported cases of AAB in society.

A large proportion of individuals exhibiting AAB report high levels of exposure to adverse childhood experiences (ACE; Widom and Maxfield, 2001; Fagan, 2005) and substantial evidence exists to support the “cycle of violence” theory in which victims later become victimizers (Widom, 1989; Widom and Maxfield, 2001; Fagan, 2005). Widom (1989) paved the way for longitudinal studies of children in monitoring how their exposure to various forms of psychosocial adversity related to subsequent exhibition of AAB in later youth and adulthood. To date, a large body of research, cross-sectional as well as longitudinal, supports this concept, and ACE is widely recognized as a precursor for a variety of negative life outcomes (Widom and Maxfield, 2001; Green et al., 2010; Klinteberg et al., 2011; Liu and Miller, 2014; Juan et al., 2020). Felitti et al. (1998) brought the negative effects of ACE to wider attention through the Adverse Childhood Experiences study, in which a large sample from the general population was surveyed on seven different psychosocial adversities, each scoring one point on the ACE scale. In the general population, 24.9% reported exposure to at least one ACE, and 6.2% reported an exposure to four or more. Odds ratios showed an increase in a variety of health risk behaviors and diseases in adulthood, such as smoking, drug use, depression, and suicide attempt for those reporting a higher number of ACE. The increased attention to the negative life outcomes from ACE led to the development of an ACE international questionnaire (ACE-IQ) with the aim to measure ACE from a global perspective (WHO, 2011). The definition of what constitutes ACE was widened to also include adversities experienced outside the immediate household such as peer violence and exposure to community violence. In a recent review of studies employing the ACE-IQ it was found that 75% in community samples worldwide reported at least one ACE (Pace et al., 2022).

Many studies have reported an association between ACE and AAB, commonly measured through crime-records, in youths and adults (Baglivio et al., 2014; Levenson and Socia, 2016; Wolff et al., 2017). Higher rates of ACE have been related to serious and persistent delinquency among youth (Fox et al., 2015) and to younger age at first arrest (Baglivio et al., 2020). A recent review by Folk et al. (2021) found an average ACE score of three in juvenile populations, and a recent meta-analysis of childhood abuse in adults incarcerated in Canadian prisons showed that 50% reported one or more ACE (Bodkin et al., 2019). In an adult sex-offender sample, Levenson and Socia (2016) found that 45% reported exposure to four or more ACE, similar to the results from Skarupski et al. (2016), who found that 50% of their adult offender sample reported exposure to four or more ACE. These incidence rates of ACE in forensic settings are interesting as the incidence rate of four or more ACE hovers around only 6% in the general population (Felitti et al., 1998).

Despite the growing number of studies on the ACE–AAB relationship, few studies have examined the phenomenon in a forensic psychiatric population. What little research does exist, however, indicates that levels of ACE in forensic psychiatric populations exceed those reported in community samples (Stinson et al., 2016, 2021). Stinson et al. (2021) used a 9-item ACE scale in their study and reported a mean ACE score of 2.6 and an ACE score of four or higher in 32% of their forensic psychiatric sample. In a previous study (2016), the authors reported that 20% of a forensic psychiatric sample had ACE scores of four or above on a 6-item ACE scale. These elevated levels of ACE in forensic psychiatric populations have been related to age at first arrest and at first physical aggression (Stinson et al., 2016) and number of violent acts (Bruce and Laporte, 2015). In sum, the research indicates that prevalence rates of ACE among forensic psychiatric populations are similar to point estimates found in other forensic populations (Fox et al., 2015; Bodkin et al., 2019).

However, findings have been inconsistent, and point estimates vary greatly between studies (Bodkin et al., 2019). These differences in results might be due to methodological variations such as the number and type of ACE items included, data extraction methods, and how the outcome measures were operationalized. Further adding to the complexity, the association between ACE and AAB seems to be reduced, even rendered insignificant, when covariates such as genetic and environmental confounders and various sociodemographic variables are accounted for (Forsman and Långström, 2012; Jung et al., 2015). Bruce and Laporte (2015), for example, found that adjusting for early onset antisocial personality disorder rendered the ACE–AAB link insignificant in a sample of forensic psychiatric patients.

Given the current state of knowledge described above, a link between ACE and AAB can be expected in forensic psychiatric samples. However, deeper knowledge on the extent of this association and its possible mechanisms is necessary. A construct of special interest here would be the propensity to act recklessly, irresponsibly, and with lack of forethought, dispositions all commonly labeled under the term “disinhibition” (Krueger et al., 2002; Young et al., 2009). The construct of disinhibition, which is conceptually close to, but distinct from impulsivity, can be understood as a coherent, highly heritable, neurobehavioral, latent dimension that confers vulnerability to externalizing psychopathology (such as for example substance abuse and interpersonal aggression; Gorenstein and Newman, 1980; Joyner et al., 2021). Disinhibition has been robustly related to AAB (de Carvalho et al., 2013; Wibbelink et al., 2017), with higher levels demonstrated in forensic psychiatric populations than in the general population (Delfin et al., 2020). Interestingly, findings from American veterans suggest that behavioral expressions of disinhibition may be moderated by the degree of exposure to traumatic life stressors (Sadeh et al., 2018) and interactions between childhood maltreatment and a disinhibited disposition has been linked to the development of subsequent externalizing problems. Whether, or how, this is applicable to forensic psychiatric populations remains largely unknown. Increased knowledge on the possible interactions among ACE, AAB, and disinhibition in forensic populations could provide important knowledge for understanding the development and persistence of AAB.

The first aim of this study was to investigate the associations between ACE, disinhibition, and AAB in a sample of forensic psychiatric patients. We expected that all study variables would be positively associated with each other. Secondly, we aimed to explore whether ACE moderated the effect of disinhibition on AAB.

## Materials and methods

### Participants

Participants were inpatients at a high-security forensic psychiatric clinic in Sweden, and data was collected from November 2016 to November 2020. The initial goal was to recruit *n* = 100 participants, but due to Covid-19 restrictions, inclusion was terminated in November 2020 when 98 participants had been included. Participants eligible for participation were those with an expected stay of more than 8 weeks who could complete the questionnaires/interviews without an interpreter. The treating psychiatrist assessed each patient before inclusion, and any patient assessed as unable to provide informed consent due to their current mental state was excluded. Of 277candidates for participation, 93 were excluded due to insufficient length of stay, insufficient language skills, or assessed inability to provide informed consent. The remaining 184 individuals who were cleared for participation by their treating psychiatrist were given information and asked to participate, of whom 83 declined participation, and three withdrew their initial consent before participation. This resulted in a sample of 98 participants. However, because nine individuals chose to terminate their participation before all data were collected, we had self-reports from a sample of *n* = 89 participants, but medical data from a sample of *n* = 98 participants (56% participation rate). The mean age of the participants was 34.9 years (range 19–62, *SD* = 10.7), and 86.7% were male (*n* = 85). The mean length of stay during their current forensic psychiatric care period was 23.5 months (range 1–135, *SD* = 33.5), with most participants (*n* = 87, 88.8%) being treated under “special care supervision,” indicating increased risk of recidivism. Only 14.3% (*n* = 14) of the participants had been subject to previous forensic psychiatric care. See Laporte et al. (2021) for a detailed clinical and psychosocial description of the sample.

### Procedure

All participants received oral and written information about the study before their participation. Subsequently, data collectors took parts of all file information, including forensic psychiatric investigations, medical records from psychiatric healthcare facilities, detailed reports on previous living circumstances and criminal histories, written court verdicts, and incidents during current incarceration that were available from the medical files. Thereafter, the participant met the data collector to complete a structured interview and answer self-report questionnaires. After all data were collected for each participant, they were assessed for quality through a review by the data collector and a senior clinician/researcher in the field. Every participant received a small monetary compensation (99 SEK) for their contribution to the study.

### Measures

#### Aggressive antisocial behaviors

AAB were measured through the Life History of Aggression questionnaire (LHA; Brown et al., 1982), consisting of 11 items reflecting different forms of AAB. The items were rated by the data collector on a 5-point scale based on the frequency of such behaviors as reported in files and interviews (0 = no events; 5 = so many events that they cannot be counted) and summed to a total scale composed of three subscales: Aggression, Self-directed Aggression, and Antisocial Behavior (Coccaro et al., 1997). The subscale Aggression includes five items that measure assault, temper tantrums, and verbal and physical aggression. The Antisocial Behavior subscale is composed by four items that measure disciplinary problems, at school and at work, and antisocial behavior with or without police involvement. The Self-directed Aggression subscale was omitted from subscale analyses in this study as its two items measure behaviors that cannot be defined as purely aggressive or antisocial. Coccaro et al. (1997) reported excellent test–retest reliability for the total LHA scale as well as the two subscales Aggression and Antisocial Behavior, with correlations in the range of 0.80–0.91. Internal consistency for the LHA total score has likewise shown excellent qualities with a Cronbach’s alpha of 0.88, and the subscales Aggression and Antisocial Behavior have shown internal consistency values of 0.87 and 0.74, respectively, (Coccaro et al., 1997). The LHA questionnaire is a well-validated instrument and has previously been used in studies of Swedish forensic psychiatric patients (Hofvander et al., 2011).

#### Disinhibition

As a measure of disinhibition, we used the Externalizing Spectrum Inventory-Brief Form (ESI-BF; Patrick et al., 2013). The ESI-BF, developed with the aim to capture a broad spectrum of externalizing psychopathology and behaviors, is a self-report questionnaire and consists of 160 items rated on a four-point Likert scale (0 = Not true at all; 3 = Completely true). The item scores are summed on three subscales: General Disinhibition (GD), Callous-Aggression, and Substance Abuse. The current study used only the 20 items in the GD subscale, giving a range of scores from 0 to 60. The GD subscale proved to have a good internal consistency in our sample, with a Cronbach’s alpha of 0.89. This is in line with previous studies reporting good psychometric properties for this subscale in the ESI-BF questionnaire (Patrick et al., 2013).

#### Adverse childhood experiences

ACE were measured in two different ways in this study: self-reported through the Childhood Trauma Questionnaire-Short Form (CTQ-SF; Bernstein et al., 2003) and clinically assessed through file reviews and interviews. The CTQ-SF assesses experiences of childhood maltreatment through 28 items rated on a 5-point Likert scale (0 = never true; 4 = very often true) and summed on five subscales: Sexual Abuse, Physical Abuse, Emotional Abuse, Emotional Neglect, and Physical Neglect. The Swedish version of the CTQ-SF used in this study demonstrated good internal consistency as measured with Cronbach’s alpha at 0.87. This is in line with previous studies reporting similar satisfying internal consistency (Gerdner and Allgulander, 2009).

The clinically assessed ACE scale was derived from file reviews, complemented by interviews to provide full information on the following variables measuring various psychosocial adversities: bullying victimization during childhood, parent(s) absent during childhood, alcohol and drug abuse in parent(s) during childhood, placement outside of family household, having witnessed violence between parents during childhood, having been exposed to physical or sexual abuse during childhood, and parental mental disorder. All nine items included in the ACE scale were dichotomized (0 = not present, 1 = present) and then computed to a total ACE score. No missing values were allowed in the computation of the clinically assessed ACE scale (*Mdn* = 3, *IQR* 2–5). The item with the highest degree of missing values regarded whether the participant had been sexually abused during childhood (*n* = 10 missing), whereas information on whether the participant had been placed outside of family home was obtained for all 98 participants. The clinically assessed ACE scale was finally based on 78 participants and showed an acceptable Cronbach’s alpha at 0.73.

### Statistical analysis

Data were anonymized and analyzed using IBM SPSS version 27. We used two-tailed *p*-values with a threshold of *p* < 0.05. Data were highly skewed for several of the dependent and independent variables. Systematically missing values were excluded from analyses. However, in cases where two items or fewer were missing, these were replaced with their serial mean for CTQ-SF, LHA, and GD.

In response to the first research question, Spearman’s rho correlations were used to investigate bivariate associations among ACE, AAB, and disinhibition. To estimate the 95% confidence interval [CI] of each coefficient, we performed bootstrapping with 1,000 samples. We used bootstrapping because it makes no assumptions and is a robust method for estimating sample parameters.

To answer the second research question, simple ordinary least squares regression was used to test whether ACE moderated the association between disinhibition and AAB. The independent variables were grand mean centered prior to computation of the interaction term. We performed centralization because it provides a more intelligible interpretation of the lower-order predictors. Since we used two measures of ACE, the CTQ-SF total scale and the clinically assessed ACE scale, two regressions models were created: one accounting for the effect of the clinically assessed ACE scale and its interaction with GD, and one accounting for the effect of the CTQ-SF and its interaction with GD. Multicollinearity was not an issue in the models considering the variance inflation factor and tolerance values presented. Residuals were normally distributed in all models.

## Results

### Associations between study variables

**Figure 1:**
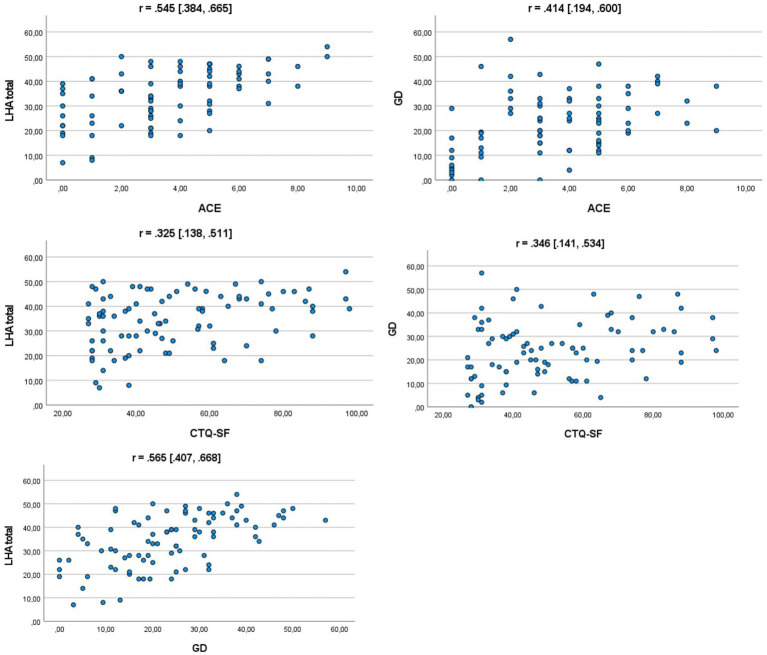
Scatterplots depicting associations between study variables. LHA total = total score on the Life History of Aggression questionnaire, CTQ-SF, Childhood Trauma Questionnaire-Short Form; ACE, Clinically assessed scale of adverse childhood experiences; GD, general disinhibition; r, Spearman’s rho with bootstrapped confidence intervals in square brackets.

Results showed that AAB was related to both ACE and disinhibition. Figure 1 provides scatterplots depicting the association between AAB (LHA), ACE (CTQ-SF and the clinically assessed ACE scale), and GD. Spearman’s rho correlations with bias corrected and accelerated bootstrap 95% CIs (in square brackets) are reported above each scatterplot. All coefficients were significant at *p* < 0.01.

### Moderation analysis

As shown in Table 1, no interaction term was found significant for either model; however, GD had a significant main effect in both models. ACE, as measured through the clinically assessed ACE scale, had a significant main effect, while CTQ-SF demonstrated no significant main effect. Each model explained 41% and 28%, respectively, of the variance in the LHA total score.

**Table 1:** Simple ordinary least squares regression testing independent and moderation effects of disinhibition, ACE, and CTQ-SF on the prediction of LHA.

	*β*	*p*	CI		*R* ^2^
Model 1					0.413
GD	0.362	0.001	0.143	0.504	
ACE	0.422	0.000	1.094	2.935	
ACE × GD	−0.008	0.928	−0.074	0.068	
Model 2					0.283
GD	0.483	0.000	0.239	0.624	
CTQ-SF	0.167	0.120	−0.025	0.213	
CTQ-SF × GD	−0.018	0.864	−0.010	0.009	

GD, general disinhibition; ACE, adverse childhood experiences; CTQ-SF, Childhood Trauma Questionnaire-Short Form; β, standardized beta coefficient; CI, 95% confidence intervals; *R*^2^, adjusted *R* squared.

### *Post hoc* analysis

**Figure 2:**
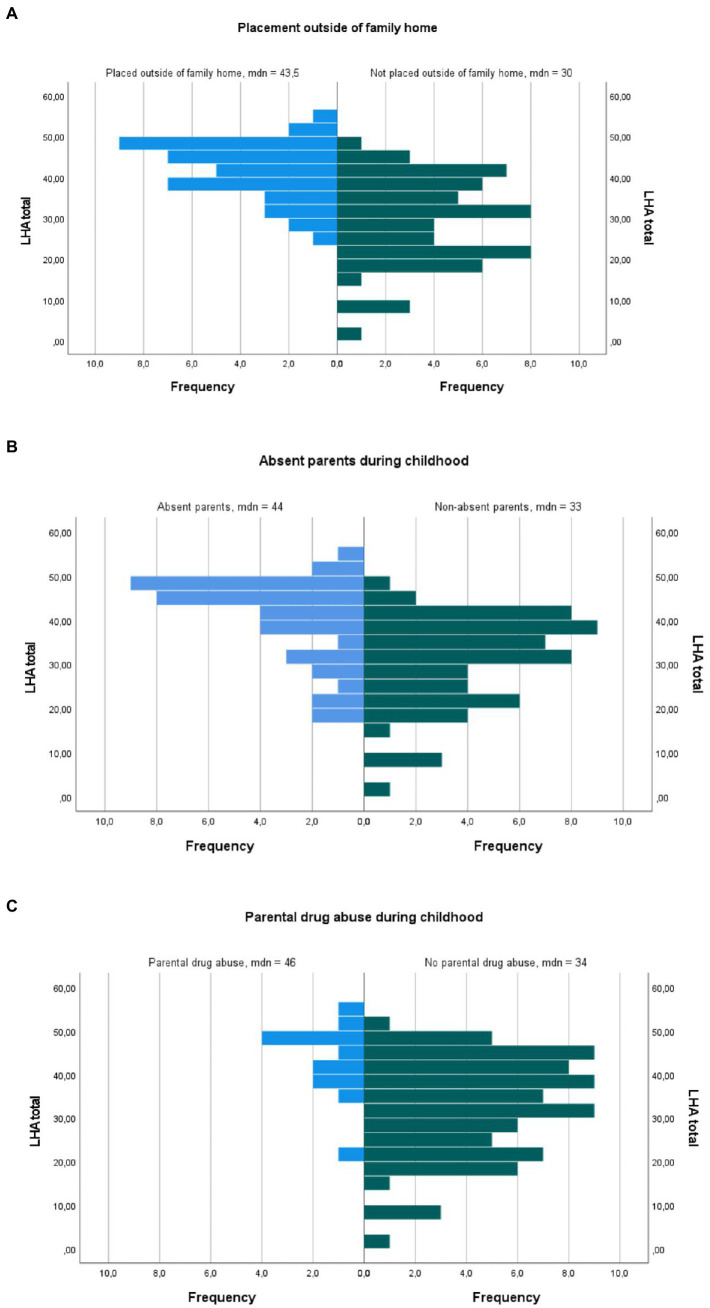
Population pyramids showing the distribution on level of AAB in three different ACE conditions. **(A)** Placement outside of family home. **(B)** Absent parents during childhood. **(C)** Parental drug abuse during childhood.

*Post hoc* analyses were performed using the Mann–Whitney test to investigate differences in outcome on LHA total score and the subscales Aggression and Antisocial Behavior for the nine different adversities composing the clinically assessed ACE scale. Results from these analyses are shown in Table 2. Assumption of homogeneity was checked for with Levene’s test and, when Levene’s test produced a *p*-value of <0.05, the distributions were checked visually. No large violation of homogeneity of variance was spotted. Results showed that LHA levels differed significantly across all adversities except having been sexually abused (*U* = 906, *z* = 1.10, *p* = 0.273, *r* = 0.12) or having been bullied (*U* = 1193.5, *z* = 1.31, *p* = 0.190, *r* = 0.14). Three adversities that stood out in the analyses through producing larger effect sizes are visualized in Figure 2. Most prominently, participants who had been placed outside of the family home had higher LHA total scores (*Mdn* = 43, *n* = 40) than those who had not (*Mdn* = 30.02, *n* = 57; *U* = 1882.5, *z* = 5.44, *p* < 0.001, *r* = 0.55). Likewise, participants who had grown up with at least one absent parent reported higher LHA scores (*Mdn* = 44, *n* = 39) than those who had had both parents present (*Mdn* = 33, *n* = 58; *U* = 1708.5, *z* = 4.25, *p* < 0.001, *r* = 0.43). Finally, participants who had grown up with parents abusing drugs reported higher LHA scores (Mdn =46.06, *n* = 13) than those with no experience of parental drug abuse (*Mdn* = 34, *n* = 77; *U* = 800.5, *z* = 3.45, *p* = 0.001, *r* = 0.36).

**Table 2:** Mann–Whitney *U* test investigating differences in LHA total score and subscales Aggression and Antisocial Behavior on the nine ACE-items included in our clinically assessed ACE scale.

	LHA total			Aggression			Antisocial behavior		
	*U*	*p*	*r*	*U*	*p*	*r*	*U*	*p*	*r*
Sexual abuse	906	0.273	0.12	917.5	0.228	0.13	839	0.632	0.05
Physical abuse	1,410	0.016	0.25	1,345	0.055	0.20	1,273.5	0.168	0.14
Bullied	1,193.5	0.190	0.14	1,074	0.720	0.04	1,059	0.810	0.06
Witnessing violence between parents	1,438.5	0.001	0.34	1,394.5	0.004	0.31	1,382.5	0.005	0.30
Parental drug abuse	800.5	0.001	0.36	745.5	0.005	0.30	757.5	0.003	0.31
Parental alcohol abuse	1,285	0.013	0.26	1,208.5	0.062	0.20	1,295.5	0.010	0.27
Placement outside of family	1,882.5	0.000	0.55	1,769	0.000	0.47	1,827	0.000	0.51
Parental mental disorder	1,227.5	0.016	0.26	1,154.5	0.072	0.19	1,099.5	0.181	0.14
Absent parents	1,708	0.000	0.43	1,668	0.000	0.40	1,656.5	0.000	0.39

*r*, effect size.

## Discussion

In this study, we found that both clinically assessed and self-reported ACE was related weakly to moderately both to disinhibition and AAB. The clinically constructed ACE scale, however, had a stronger association to disinhibition and AAB, with a clear main effect in the regression model, while self-reported ACE, although significantly related in the bivariate analyses, showed no significant main effect in the regression model. No moderating effect of ACE was found on the association between disinhibition and AAB in this sample for either ACE measure. *Post hoc* analyses showed that various adversities affected LHA scores differently; placement outside the family and absent parents during childhood produced the largest effect sizes, indicating that these adversities had a relatively larger impact on levels of AAB than other types of ACE included in the study.

Our results showed that ACE were positively related to AAB and disinhibition in a forensic psychiatric context. This is in line with previous findings from forensic psychiatric samples (Stinson et al., 2016). Our patients reported disproportionately high levels of ACE, indicating that ACE and disinhibition are both clinically relevant constructs in understanding aggressive antisocial behaviors. The findings in the correlational analysis suggest that in our pursuit of understanding and eventually preventing AAB, we should pay more attention to these individuals’ exposures to ACE. Such consideration may be especially challenging in working with individuals exhibiting AAB, who may elicit anger and fear in care staff. However, from a therapeutic point of view, this special group of individuals warrants trauma-informed psychiatric care.

To the best of our knowledge, few studies have used a combination of self-report measures and file reviews to measure ACE, and some interesting differences between the measures are apparent. The self-report questionnaire CTQ-SF showed a weaker association of ACE with both disinhibition and AAB than the clinically assessed ACE scale. These differences partly reflect differences in data extraction and how ACE is measured in the current study. Since CTQ-SF is a self-report measure it conveys to a larger extent participants’ subjective experiences of their upbringing compared to the items included in the clinically assessed ACE scale. However, the clinically assessed ACE scale does to a larger extent than the CTQ-SF include ACE items outside of family household such as bully victimization and placement outside of family home. Including such adversities in measuring ACE is in line with previous reviews on ACE measures that have called for a widening of the ACE concept to also include adversities that take place outside of the immediate household of upbringing (Pace et al., 2022).

Several explanations may exist to explain differences in results in our two measures of ACE. It is possible that individuals growing up in dysfunctional and abusive household acclimatize to elevated levels of violence, distress, and neglect, and this may be reflected in the weaker associations between CTQ-SF and AAB. On a similar note, previous studies support the notion that false negatives are common in retrospective measures of ACE (Hardt and Rutter, 2004) and that errors are frequent in surveys posing sensitive questions such as exposure to sexual victimization during childhood (Tourangeau and Yan, 2007). Such aspects may deflate estimates of self-reported ACE. Furthermore, our ACE scale included placement outside the family home, which has not conventionally been a component in previous composite ACE scales. However, placement outside of family home has previously proven to be a strong predictor of psychiatric hospitalization and aggressive behaviors in forensic psychiatric samples (Stinson et al., 2016, 2021).

In our sample, placement outside of the family home was the most prominent ACE as measured by its impact on levels of AAB. Several explanations may exist to account for this. The finding could partly or wholly be explained by conduct problems in childhood and youth among our participants. This would be in line with findings by Bruce and Laporte (2015) who found the ACE-AAB link rendered insignificant when adjusting for early onset antisocial personality disorder. It is also possible that placement outside the family home serves as an indicator of the severity and pervasiveness of a dysfunctional and abusive household, which may not otherwise be easily assessed in file reviews (Stinson et al., 2021). It is likewise possible that placement outside the family home in itself may contribute to a negative developmental trajectory characterized by high levels of AAB. This is in line with findings by Sarisaslan et al. (2022) who found that placement outside of the family home increased risks for subsequent negative life outcomes, including antisocial behavior, even after adjusting for a variety of measured and unmeasured confounders, including preplacement factors.

Placement outside the family home proved to be the strongest ACE in our clinically assessed scale to differentiate individuals by their levels of AAB. It may be that for individuals who report high levels of exposure to various forms of ACE, other adversities than those assessed in the original ACE survey (Felitti et al., 1998) and currently in use in the ACE-IQ (WHO, 2011) are important. Critics of the ACE-IQ have pointed to a lack of cultural sensitivity in measuring ACE globally (Quinn et al., 2018). Being sensitive to the culture and context within which ACE is measured meanwhile not needing to reinvent the ACE concept in each specific sample remains a challenge for research and clinicians alike. ACE items included in future research on this specific group of individuals should be chosen carefully and we suggest that broadening the ACE concept to include adversities outside of the immediate household such as out-of-home placement may be especially important in a forensic psychiatric context.

The three ACE with the largest effect on levels of AAB in this study group produced, individually, an increase of approximately one standard deviation, equal to a one-point increase on all variables for AAB, on the LHA questionnaire. It is clear that forensic psychiatric patients represent a vulnerable group with both self-reported and documented exposure to a variety of ACE. Previous research has shown a dose–response relationship between ACE and AAB; however, our results show that some ACE matter more than others in terms of AAB scores. Considering that placement outside of family home produced an increase of over one standard deviation on AAB levels, future research should aim to identify how such an intervention may be better tailored and implemented.

No moderating effect of ACE was found on the GD–AAB relationship. It may be that other personality-related constructs measured through other instruments than the ESI-BF might interact with ACE on AAB outcomes. It is also worth mentioning that ACE scores are not deterministic. Not all individuals who experience ACE go on to exhibit AAB and there may exist mediators, not yet known, between exposure to ACE and negative life outcomes, such as AAB (Ports et al., 2020). In a study by Sadeh et al. (2018), PTSD symptoms moderated the association between disinhibition and recent risky and self-destructive behaviors in a psychiatric, non-forensic sample. Evidence has been presented supporting a mediating role of PTSD symptoms on the ACE-violence relationship in forensic and community samples (Wolfe et al., 2004; Swopes et al., 2013). It is possible that rather than adversities *per se*, it is their effect on intrapsychic processes, as shown in PTSD symptomatology, that moderates the GD–AAB relationship.

However, what stands out in our regression analyses is the rather large proportion of explained variance when ACE and GD were entered into the model. Together, they accounted for 41% of the variance in the outcome on LHA. This shows that ACE and GD may be of high clinical relevance in understanding AAB and that forensic psychiatric settings could benefit from a trauma-informed perspective in the day-to-day care of these patients.

## Limitations

Some caution should be taken in interpreting the results of this study. First, the number of *post hoc* analyses conducted in this study increase the risk for Type I error. However, even with Bonferroni-adjusted significance levels, several Mann–Whitney *U*-tests would remain significant. Second, it is worth reiterating the nature of the ACE scores and their shortcomings. Since ACE items were coded dichotomously, they neglected the frequency, intensity, and duration of the adversity in question. The measure is also agnostic as to what type of adversity might impact the outcome to a greater or a lesser degree, assigning the same quantitative importance to each type of adversity. This is not supported by our data, however, which show that different forms of maltreatment affect levels of AAB differently. Third, issues pertaining to generalizability should be noted. National legislation and Swedish court procedures may hamper generalizability to other countries forensic psychiatric populations. Furthermore, our sample consisted mainly of male adults, making it difficult to generalize our results to female forensic psychiatric patients. However, considering that most violent crimes are committed by male adults, our sample is representative of the larger forensic population in Sweden. Also, while the 56% participation rate could be considered modest, however, this sample represents about 5% of the total population of forensic psychiatric patients in Sweden. Moreover, this study was carried in a high-security facility which provides care for severely mental ill individuals referred from other clinics, which inevitably makes the recruitment even more difficult than have already been described by other researchers (Pedersen et al., 2021). Although previous studies have reported gender differences in exposure to ACE indicating that women are exposed to higher levels of ACE than men (Stinson et al., 2016; Giarratano et al., 2020), it may be that justice-involved, mentally ill women represent a unique group in the forensic psychiatric context, with different needs and other psychosocial adversities playing a different role in their development of AAB. Future research should be wary of this, and the choice of ACE items should be made in consideration of the sample composition. Overall, what is lost in generalizability in this study is compensated for by its clinical relevance. Forensic psychiatric populations represent a unique group that suffer from severe mental illness as well as life-long involvement in the criminal justice system. Prevalence rates and the cumulative effect of ACE have been understudied in this group.

## Conclusion

Our findings highlight the potential benefits in implementing a trauma-informed care in forensic psychiatric settings. Previous studies exist to support the benefits of trauma-informed care in forensic settings (Elwyn et al., 2015) and a great deal of research addresses the specific challenges in implementing such a care in forensic settings (Miller and Najavits, 2012; Levenson and Willis, 2019). Our sample reported exposures to ACE that far exceed those found in the general population (Felitti et al., 1998). Despite the cross-sectional design, our findings substantiate previous research supporting the notion of a cycle of violence in forensic psychiatric patients (Stinson et al., 2016, 2021). It is likely that maltreatment and developmental adversities alter cognitive schemas, contribute to deficits in self-management, and interfere with the ability to form healthy interpersonal relationships. Trauma-informed staff might help these patients establish and maintain healthy interpersonal relationships and, to a larger extent, engage in their psychiatric care (Levenson and Willis, 2019). Our *post hoc* analyses clearly show that different forms of maltreatment should not be assumed to be equal. Although the cumulative effect of ACE is well established and supported in our results, certain types of maltreatment, especially placement outside the family home, tend to have a larger effect on levels of AAB. Future research should aim to find adversities of pivotal importance and disentangle how such adversities affect AAB. Considering that previous research has found that placement outside of the family home increases subsequent adult criminality (Sarisaslan et al., 2022), a replication of our study in at-risk youth, placed in foster care home or in residential care, may be valuable in validating the relationships found in this study and help develop better preventive measures for individuals exhibiting AAB.

## Data availability statement

The raw data supporting the conclusions of this article will be made available by the authors, without undue reservation.

## Ethics statement

The studies involving human participants were reviewed and approved by Research Ethics Committee at Linköping University, 2016/213-31 and 2017/252-32. The patients/participants provided their written informed consent to participate in this study.

## Author contributions

AM and MW developed the study design. Data collection was performed by NL and JB. AM conducted data analyses and drafted the paper. MW, JB, and NL provided critical revisions. All authors contributed to the article and approved the submitted version.

## Funding

This work was supported by FoU Kronoberg and the Swedish Research Council for Health, Working Life and Welfare (2018-01409).

## Conflict of interest

The authors declare that the research was conducted in the absence of any commercial or financial relationships that could be construed as a potential conflict of interest.

## Publisher’s note

All claims expressed in this article are solely those of the authors and do not necessarily represent those of their affiliated organizations, or those of the publisher, the editors and the reviewers. Any product that may be evaluated in this article, or claim that may be made by its manufacturer, is not guaranteed or endorsed by the publisher.
